# Glutamate-stimulated peroxynitrite production in a brain-derived endothelial cell line is dependent on *N*-methyl-d-aspartate (NMDA) receptor activation

**DOI:** 10.1016/j.bcp.2006.09.021

**Published:** 2007-01-15

**Authors:** G.S. Scott, S.R. Bowman, T. Smith, R.J. Flower, C. Bolton

**Affiliations:** a Centre of Biochemical Pharmacology & Experimental Pathology, The William Harvey Research Institute, St. Bartholomew's Hospital Medical College and the London School of Medicine and Dentistry, Charterhouse Square, London EC1M 6BQ, United Kingdom; b NeuMatRx, Truro, Cornwall TR3 6NT, United Kingdom

**Keywords:** Peroxynitrite, Nitric oxide, Glutamate, *N*-Methyl-d-aspartate receptor, Brain-derived endothelial cells

## Abstract

There is accumulating and convincing evidence indicating a role for glutamate in the pathogenesis of the human demyelinating disease multiple sclerosis (MS). Studies in experimental autoimmune encephalomyelitis (EAE), the animal model of MS, demonstrate that pharmacological inhibition of specific glutamate receptors suppresses neurological symptoms and prevents blood–brain barrier (BBB) breakdown. The mechanisms through which glutamate influences BBB function during EAE remain unclear. Glutamate triggers the production of nitric oxide and superoxide, which can lead to the formation of peroxynitrite (ONOO^−^). Recent studies have implicated ONOO^−^ in the loss of neurovascular integrity during EAE. We propose that glutamate contributes to BBB breakdown via the actions of ONOO^−^. The present investigation examined glutamate-induced ONOO^−^ formation in the b.End3 brain-derived endothelial cell line. b.End3 cells were incubated with a concentration range of glutamate and ONOO^−^ production was assessed over time. Results showed a concentration- and time-dependent increase in ONOO^−^ levels in glutamate-treated cells that were suppressed by selective and non-selective inhibitors of ONOO^−^-mediated reactions. Specific activation of b.End3-associated NMDA receptors also resulted in a concentration-dependent increase in ONOO^−^ production. The ability of b.End3 cells to respond to the presence of glutamate was confirmed through the detection of NMDA receptor immnuoreactivity in cell extracts. In addition, the use of the NMDA receptor antagonists MK-801 and memantine reduced glutamate-mediated ONOO^−^ generation from b.End3 cells. The data reinforce the important relationship between glutamate and the NMDA receptor, positioned at neurovascular sites, which may be of particular relevance to the pathogenesis of demyelinating disease.

## 1 Introduction

Multiple sclerosis (MS) is a human neurodegenerative disorder of unknown aetiology. There is growing evidence that the excitatory amino acid glutamate has an important role in the pathogenesis of MS [1–3]. Glutamate concentrations are increased in cerebrospinal fluid from MS patients and the levels correlate with the severity of disease [4,5]. Alterations in the metabolism and transport of glutamate have been identified in MS patients and changes to the balance of glutamate in the central nervous system (CNS) have been associated with local tissue damage [6–8]. Related work in the animal model of MS, experimental autoimmune encephalomyelitis (EAE), strongly implicates glutamate in disease development [9–11]. In particular, *N*-methyl-d-aspartate (NMDA) and α-amino-3-hydroxy-5-methyl-4-isoxazolepropionic acid (AMPA) receptor antagonists have been shown to suppress EAE, and inhibition of glutamate transmission also reduces disease severity [12–16].

There may be many pathways through which glutamate mediates deleterious effects in MS and EAE but the ability of glutamate to induce CNS cell death has gained particular attention [17]. Indeed, glutamate-induced excitotoxicity is thought to contribute to oligodendrocyte and axonal loss in MS and EAE and the amino acid also exerts toxic effects on neurons [8,14,18–21]. Several studies have proposed glutamate involvement in the pathogenesis of MS and EAE through altering blood–brain barrier (BBB) integrity. The BBB assists in the maintenance of homeostasis in the CNS [22] and abnormal neuroendothelial function during MS and EAE allows excess movement of fluid and cells into brain and spinal tissues [23,24]. Several studies have shown that glutamate directly modulates neurovascular integrity [25–27] and administration of glutamate receptor antagonists restricts BBB breakdown during EAE [12,13].

The mechanisms through which glutamate modifies neurovascular integrity are unclear but have been shown to involve the vasoactive molecules nitric oxide (NO) and superoxide (O_2_^−^) that can combine to form damaging levels of peroxynitrite (ONOO^−^) [25,28–30]. Indeed, glutamate is known to stimulate the production of NO and O_2_^−^ and has the capacity to generate ONOO^−^ in the CNS [31]. Therefore, glutamate has the potential to mediate BBB breakdown in MS and EAE via the actions of ONOO^−^.

The current study employed the brain endothelial cell line, b.End3, to investigate the relationship between glutamate-mediated NMDA receptor activation and ONOO^−^ production in neuroendothelial breakdown. The work confirms b.End3 cells generate ONOO^−^ following exposure to glutamate. In particular, the investigation suggests glutamate-mediated ONOO^−^ production by b.End3 cells occurs through NMDA receptor activation and NO production via up-regulation of specific NO synthase activity.

## 2 Materials and methods

### 2.1 Chemicals and antibodies

*N*^G^-Nitro-l-arginine methyl ester (l-NAME), *N*^G^-monomethyl-l-arginine (l-NMMA), l-*N*^5^-(1-iminoethyl)ornithine (l-NIO), *N*-acetyl-l-cysteine (*N*-AC) and *N*-(3-(aminomethyl)benzyl) acetamidine (1400W) were obtained from Alexis Corporation, Nottingham, UK. MK-801 (dizocilpine maleate, (5*R*,10*S*)-(+)-5-methyl-10,11-dihydro-5H-dibenzo[a,d]-cyclohepten-5-10-imine maleate), memantine (3,5-dimethyl-tricyclo [3.3.1.13,7] decan-1-amine hydrochloride), 2,3-dioxo-6-nitro-1,2,3,4-tetrahydrobenzo-[f]quinoxaline-7-sulphonamide (NBQX), (*RS*)-α-cyclopropyl-4-phosphonophenylglycine (CPPG) and *N*-phenyl-7-(hydroxyimino) cyclopropa[b] chromen-1a-carboxamide (PHCC) were purchased from Tocris Cookson Ltd, Bristol, UK. Anti-mouse NMDAR_1_ and NMDAR_2A/B_ polyclonal antibodies were purchased from Chemicon International, Hants, UK. Enhanced chemiluminescence (ECL) reagent was obtained from Amersham Biosciences, Bucks, UK. Peroxidase conjugated goat anti-rabbit antibody and uric acid (UA) were purchased from Calbiochem, Nottingham, UK.

### 2.2 Culture of b.End3 cells

The SV129 mouse brain endothelial cell line b.End3 (European Collection of Animal Cell Cultures, Wiltshire, UK), established using the Polyoma virus middle T-antigen [32], was maintained in Dulbecco's modified Eagle's medium (DMEM) supplemented with 2 mM l-glutamate, 5 μM mercaptoethanol, 1% non-essential amino acids, 10% fetal bovine serum, 1 mM sodium pyruvate, 50 U/ml penicillin and 50 μg/ml streptomycin (Invitrogen Ltd, Paisley, Scotland, UK).

### 2.3 Cell treatment

b.End3 cells were cultured in 96 well plates at a density of 5 × 10^4^ cells/ml. Confluent cell preparations were incubated with a range of glutamate concentrations for 1, 4 and 24 h to determine ONOO^−^ and NO production and assess cell viability. In addition, b.End3 cells, at an original density of 2.5 × 10^5^ cells/ml, were cultured to confluence in 24 well plates and exposed to glutamate in the presence and absence of UA, selective NOS inhibitors and glutamate receptor antagonists. The generation of ONOO^−^ by b.End3 cells was then assessed at pre-determined time points.

### 2.4 Cell viability

The mitochondrial-dependent reduction of 3-[4,5-dimethylthiazol-2-yl]-2,5-diphenyl-tetrazolium (MTT) to formazan was used as an indicator of cell viability. Cells in 96-well plates were exposed to glutamate concentrations from 0.001 mM to 20 mM for 1, 4 or 24 h followed by the addition of MTT, at 0.2 mg/ml, to the cultures. After 1 h incubation at 37 °C, supernatants were removed and the cells lysed by the addition of 100 μl aliquots of dimethyl sulphoxide to each well. The reduction of MTT to formazan within cells was measured using a Spectramax 250-microplate reader (Biotek EL310) at 550 nm absorbance. Results are expressed as percentage (%) viability compared to untreated controls.

### 2.5 Measurement of NO production

The generation of NO was assessed by measuring nitrite, the co-principle, stable end-product of NO metabolism, in cell supernatants. The assay, developed from a previous method [33] quantified nitrite by adding an equal volume of Griess reagent to each cell supernatant and, after incubation at room temperature for 10 min, the absorbance of each sample was read at 550 nm using a microplate reader (Spectramax 250, Biotek EL310). The nitrite level in samples was determined by reference to a series of sodium nitrite standards prepared in culture medium. Results are presented as μM nitrite/100 μl cell supernatant.

### 2.6 Measurement of ONOO^−^ formation

The formation of ONOO^−^ was determined by measuring the ONOO^−^-dependent oxidation of dihydrorhodamine (DHR) 123 to rhodamine 123 by b.End3 cells. Following glutamate treatment cell preparations were rinsed with phosphate buffered saline and incubated with 5 μM DHR 123 for 1 h at 37 °C. The fluorescence of rhodamine 123 was measured at excitation 485 nm, emission 530 nm using a fluorescent plate reader (Cytofluor II, Perseptive Biosystems). Results are expressed as % oxidation compared to untreated control cultures.

### 2.7 Western blot analysis of b.End3 cell preparations

Whole cell lysates were prepared from untreated b.End3 cells using Laemmli's buffer. Each sample, containing 30 μg protein, was separated on 10% SDS-PAGE gels and transferred to nitrocellulose membranes using semi-dry transfer techniques. Membranes were blocked with 10% milk protein and incubated overnight at 4 °C with 1:250 dilution of rabbit anti-mouse NMDAR_1_ or NMDAR_2A/B_ polyclonal antibody. The membranes were washed, incubated for 1 h in 1:2000 dilution of peroxidase conjugated goat anti-rabbit antibody and the protein bands visualized using ECL reagent.

### 2.8 Statistical analysis

All values are expressed as the mean ± S.E.M. of *n* observations, where *n* > 6 from at least three independent experiments. Data sets were analysed by one-way analysis of variance (ANOVA) followed by post hoc Dunnett's test. In all tests, *p* < 0.05 was considered significant.

## 3 Results

### 3.1 Effects of glutamate on b.End3 cell viability

**Fig. 1:**
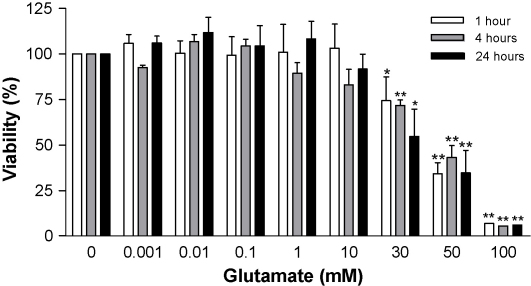
Viability of b.End3 cells exposed to glutamate. b.End3 cells were treated with varying concentrations of glutamate for 1, 4, and 24 h. Cell viability was measured by the mitochondrial-dependent reduction of MTT to formazan. Results are presented as % viability compared to untreated cultures. ^*^p < 0.05 and ^**^p < 0.01 compared to control group by one-way ANOVA with post hoc Dunnet's test.

Normal physiological levels of glutamate in CNS cells are less than 3 mM but, during disease and injury, the interstitial fluid concentration can rise dramatically [34]. The precise concentrations of glutamate in the CNS during MS and EAE are unknown but elevations above normal levels have been reported [5,6,35]. Glutamate, at millimolar concentrations, is known to exert toxic effects on CNS-derived preparations, including cells isolated from neuroendothelial tissues [27,36]. Therefore, initial experiments were undertaken in b.End3 cells to establish a glutamate concentration that did not affect cell viability but induced ONOO^−^ release. The cells were incubated in the presence of glutamate, at concentrations from 1 μM to 100 mM, for 1, 4 and 24 h and cell viability was determined by assessing mitochondrial respiration. Glutamate levels between 1 μM and 10 mM did not affect viability in b.End3 cells over a 24 h period (Fig. 1). In contrast, concentrations of glutamate between 30 mM and 100 mM were associated with significant reductions in cell viability.

Glutamate may influence cell survival by altering the pH of the culture conditions. The pH of the media was determined after the addition of glutamate and was closely maintained at pH 7.4 up to a concentration of 20 mM (data not shown). Therefore, subsequent dose response experiments, to establish a level of glutamate which influenced NO and ONOO^−^ production, were conducted using a maximum glutamate concentration of 20 mM.

### 3.2 Glutamate-induced NO and ONOO^−^ production by b.End3 cells

**Fig. 2:**
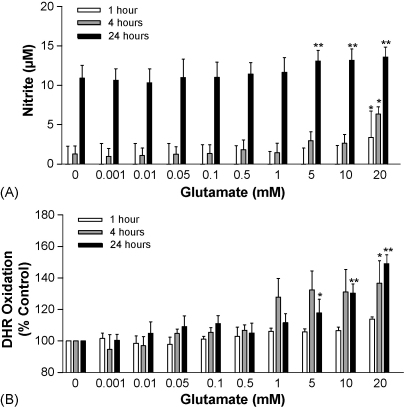
NO and ONOO^−^ production by b.End3 cells exposed to glutamate. b.End3 cells were treated with varying concentrations of glutamate for 1, 4, and 24 h. (A) NO production was measured as the nitrite content (μM) of cell culture supernatants using the Griess assay and (B) ONOO^−^ production was determined by measuring the oxidation of dihydrorhodamine (DHR) to produce the fluorescent rhodamine. Results are presented as % increase in DHR oxidation compared to untreated cultures. ^*^p < 0.05 and ^**^p < 0.01 compared to control group by one-way ANOVA with post hoc Dunnet's test.

The production of NO, measured as nitrite, and ONOO^−^, quantified by DHR oxidation, in b.End3 preparations, after exposure to increasing concentrations of glutamate, are detailed in Fig. 2A and B. Nitrite levels remained unchanged in cells after incubation, for 1–24 h, with glutamate at concentrations from 0.001 mM to 1 mM (Fig. 2A). Treatment of b.End3 cells with 5 mM to 20 mM glutamate, caused a significant increase in nitrite levels at 24 h. Moreover, 20 mM glutamate induced a significant and sustained elevation in nitrite concentrations from preparations incubated for 1 h. The production of ONOO^−^ revealed a similar profile to nitrite release after treatment of b.End3 cells with glutamate (Fig. 2B). Incubation of cells with 5 mM to 20 mM glutamate elicited a significant dose-dependent increase in ONOO^−^ synthesis. Furthermore, 20 mM glutamate raised DHR oxidation levels 4 h post-incubation.

Data from the preceding experiments confirmed that exposure of b.End3 cells to 10 mM glutamate induced a significant, reproducible and non-cytotoxic increase in ONOO^−^ production. Therefore, the supra-physiological concentration of 10 mM glutamate was used to characterise reactive nitrogen species production and NMDA receptor activation by b.End3 cells.

### 3.3 Inhibition of glutamate-induced ONOO^−^ production

**Fig. 3:**
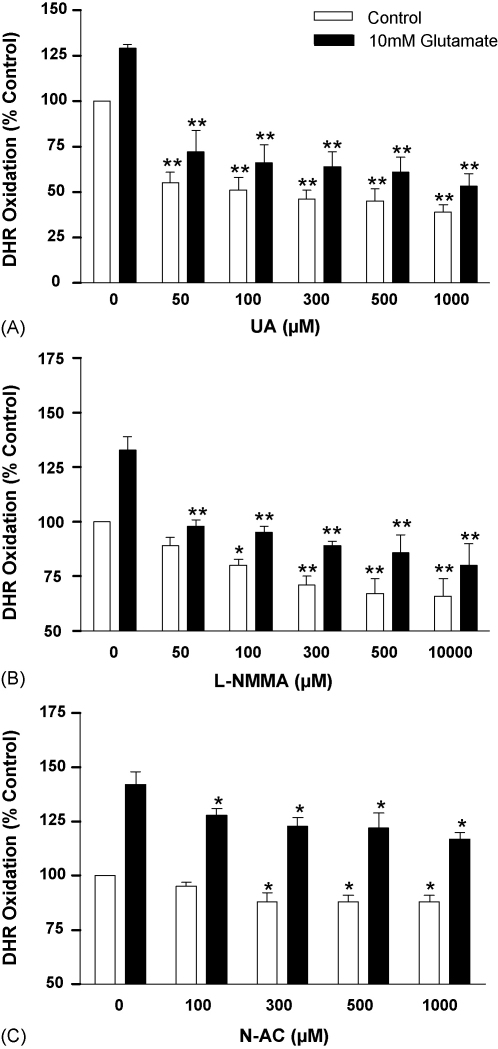
The effect of free radical inactivation, the blockade of NO production and anti-oxidants on glutamate-induced ONOO^−^ formation by b.End3 cells. Cells were exposed to 10 mM glutamate for 24 h in the presence of increasing concentrations of: (A) UA, (B) l-NMMA and (C) *N*-AC. ONOO^−^ production was measured by the oxidation of dihydrorhodamine (DHR) to produce the fluorescent rhodamine. Results are presented as % increase in DHR oxidation compared to untreated cells. ^*^p < 0.05 and ^**^p < 0.01 compared to control group by one-way ANOVA with post hoc Dunnet's test.

The decomposition of ONOO^−^, formed as a consequence of NO and O_2_^−^ interaction, generates highly reactive intermediates that can be inactivated by UA, a selective scavenger of ONOO^−^-dependent radicals [28,29]. DHR oxidation in unstimulated b.End3 cells was significantly reduced by the addition of increasing UA concentrations (*p* < 0.01) (Fig. 3A). Similarly, each dose of UA significantly inhibited DHR oxidation in glutamate-stimulated cultures (*p* < 0.01). The inhibitory effects of UA on basal and induced DHR oxidation in b.End3 cells strongly indicate that the major oxidising species present was ONOO^−^. The competitive, irreversible, pan-NOS inhibitor l-NMMA significantly reduced basal and glutamate-induced DHR oxidation in b.End3 cells (*p* < 0.01) (Fig. 3B). The addition of *N*-AC, a precursor of the anti-oxidant glutathione, to unstimulated and glutamate-exposed cultures generated a modest and significant inhibition of DHR oxidation (*p* < 0.05) (Fig. 3C).

### 3.4 Receptor types mediating glutamate-stimulated ONOO^−^ production

**Fig. 4:**
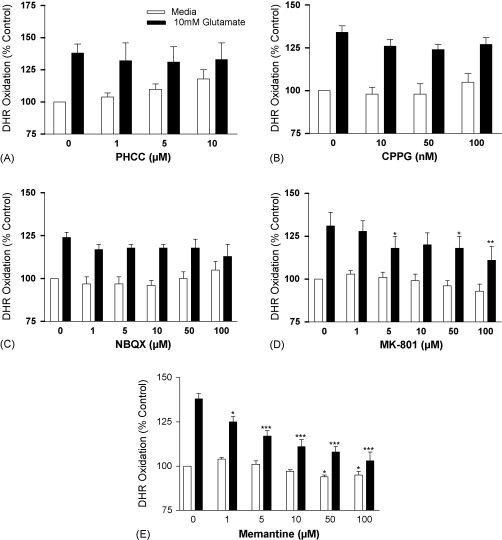
The effect of glutamate receptor antagonists on glutamate-induced ONOO^−^ production by b.End3 cells. Cells were exposed to 10 mM glutamate for 24 h in the presence of increasing concentrations of: (A) PHCC, (B) CPPG, (C) NBQX, (D) MK-801 and (E) Memantine. ONOO^−^ production was measured by the oxidation of dihydrorhodamine (DHR) to produce the fluorescent rhodamine. Results are presented as % increase in DHR oxidation compared to untreated cultures. ^*^p < 0.05, ^**^p < 0.01 and ^***^p < 0.001 compared to control group by one-way ANOVA with post hoc Dunnet's test.

Initiation of a cellular response to glutamate requires the activation of the ionotropic, NMDA and AMPA/kainate receptors, or metabotropic receptors [37]. Several antagonists were employed to determine the receptor type involved in glutamate-induced ONOO^−^ production from b.End3 cells. The addition of increasing concentrations of PHCC, a potent and selective group I metabotropic glutamate receptor antagonist, to unstimulated b.End3 cells elicited a detectable, but not significant, increase in DHR oxidation (Fig. 4A). The compound did not alter DHR oxidation in cultures exposed to glutamate. CPPG, a group II/III metabotropic receptor antagonist, had no effect on DHR oxidation in unstimulated or glutamate-stimulated b.End3 cells (Fig. 4B). Similarly, NBQX, a competitive AMPA/kainate receptor antagonist, did not alter basal or glutamate-induced responses in the cultures (Fig. 4C). In contrast, the selective, non-competitive NMDA ionotropic glutamate receptor antagonist, MK-801 elicited a modest inhibition of DHR oxidation in unstimulated b.End3 cells and significant changes in glutamate-rich cells (Fig. 4D), (*p* < 0.05–0.01). High concentrations of memantine, a relatively low potency, uncompetitive NMDA receptor antagonist that targets the channel pore, significantly suppressed DHR oxidation in unstimulated cultures (*p* < 0.05) (Fig. 4E). In particular, each dose of the drug added to glutamate-stimulated cultures significantly inhibited DHR oxidation (*p* < 0.05–0.001).

### 3.5 Glutamate receptor expression on b.End3 cells

**Fig. 5:**
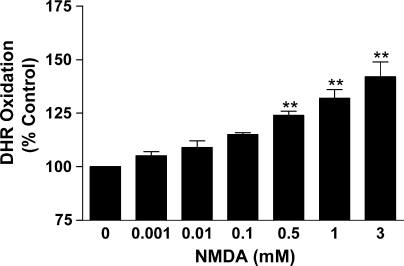
The effect of NMDA on ONOO^−^ production by b.End3 cells. Cells were incubated with increasing concentrations of NMDA for 24 h. ONOO^−^ production was determined by measuring the oxidation of dihydrorhodamine (DHR) to produce the fluorescent rhodamine. Results are presented as % increase in DHR oxidation compared to untreated controls. ^**^p < 0.01 compared to control group by one-way ANOVA with post hoc Dunnet's test.

**Fig. 6:**
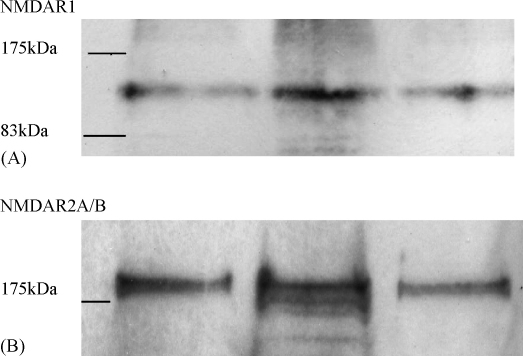
The presence of NMDA Receptor 1 (A) and NMDA receptor 2A/B (B) in protein extracts from b.End3 cells subjected to Western blotting techniques. Each lane represents whole cell extracts from three individual, untreated b.End3 cultures.

Collectively, the results provide compelling evidence that glutamate-stimulated ONOO^−^ production by b.End3 cells is mediated through NMDA receptor involvement. Further evidence indicating the presence of the receptor on b.End3 cells is provided by the dose-dependent production of ONOO^−^ following exposure of the cultures to the specific receptor ligand NMDA (Fig. 5). The location of NMDA receptors on b.End3 cells was also confirmed by incubation of separated protein extracts, from individual cultures, with specific and selective antibodies against the NMDA R1 and NMDA R2A/B receptor (Fig. 6A and B). Previous investigations have confirmed the NMDA R1 and NMDA R2A/B receptor to have molecular weights of approximately 125 kDa and 180 kDa, respectively [38,39] which corresponds with the values recorded in the current study.

### 3.6 NOS isoforms involved in the b.End3 cell response to glutamate

**Fig. 7:**
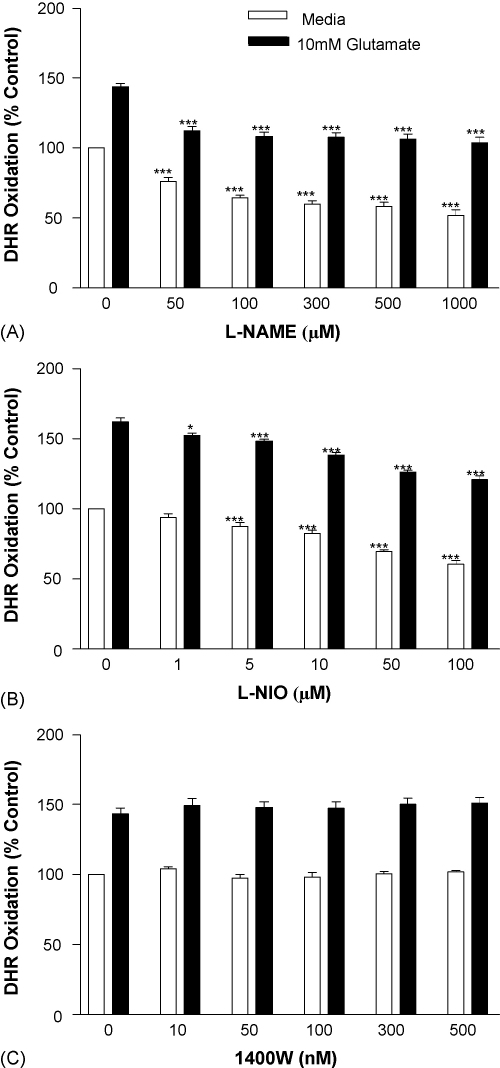
The effect of NOS inhibition on glutamate-induced ONOO^−^ production by b.End3 cells. Cells were exposed to 10 mM glutamate for 24 h in the presence of increasing concentrations of: (A) l-NAME, (B) NIO and (C) 1400W. ONOO^−^ production was determined by measuring the oxidation of dihydrorhodamine (DHR) to produce the fluorescent rhodamine. Results are presented as % increase in DHR oxidation compared to untreated cells. ^*^p < 0.05, ^***^p < 0.001 compared to control group by one-way ANOVA with post hoc Dunnet's test.

The involvement of NOS isoforms in glutamate-stimulated ONOO^−^ production by b.End3 cells was examined using specific inhibitors at concentrations previously shown to alter NOS function *in vitro* [40,41]. DHR oxidation was significantly inhibited in unstimulated and glutamate-exposed cultures at all concentrations of the pan-NOS inhibitor l-NAME (Fig. 7A) (*p* < 0.001). The endothelial (e) NOS inhibitor l-NIO also significantly suppressed DHR oxidation in b.End3 cells in the presence and absence of glutamate (Fig. 7B) (*p* < 0.05 or 0.001) but the inducible (i) NOS inhibitor, 1400W [42] (Fig. 7C), was without effect.

## 4 Discussion

The current study has demonstrated that the brain endothelial cell line, b.End3, releases ONOO^−^ and NO in the presence of a pre-determined, supra-physiological concentration of glutamate. In particular, ONOO^−^ generation, as quantified by DHR production, was significantly attenuated by the selective scavenger UA, the pan-NOS inhibitors l-NAME and l-NMMA, the anti-oxidant glutathione precursor *N*-AC and the eNOS inhibitor l-NIO. Moreover, the investigation strongly suggests the release of ONOO^−^ by b.End3 cells, in response to glutamate, is mediated via the NMDA receptor and not through AMPA/kainate receptor involvement.

BBB disruption is a cardinal sign of new and active lesions in MS but the pathways leading to neuroendothelial breakdown are not understood despite the identification of several candidate mediators [23,43–45]. Emerging evidence strongly implicates a relationship between glutamate, NMDA activation and reactive nitrogen species in the pathogenesis of MS and the related model, EAE [1,9,46]. In particular, the identified mediators have been suggested as important participants in causing loss of BBB integrity during MS and EAE [12,13,29,47].

The b.End3 cell line used in the present study has been shown in previous investigations to retain many of the characteristics of primary endothelial cells. For example, the cultures express endothelial-specific proteins including PECAM-1, endoglin, MECA-32 and Flk-1 and respond to inflammatory cytokines with the up-regulation of adhesion molecules such as ICAM-1, VCAM-1 and E-selectin and the S100 chemotactic protein CP-10 [48–54]. Furthermore, transformed cells, in contrast to non-immortalized cultures, display cytoplasmic localisation of the tight junction-associated proteins, occludin and zona occludin-1 and express iNOS and eNOS [55,56]. The results from our work further characterise the cell line by demonstrating the basal release of ONOO^−^ and NO and, in particular, the significant production of both species in response to the excitotoxic mediator glutamate.

In order to investigate the mechanisms through which glutamate induces ONOO^−^ and NO production from b.End3 cells a variety of compounds that interfere with the possible pathways to generation of the free radicals were employed. Moreover, a selection of the drugs used have relieved the neurological deficits and prevented BBB breakdown in models of EAE. For example, the selective ONOO^−^ scavenger UA has been successfully used to inhibit EAE and limit permeability changes in endothelial cells *in vitro* [28,29,47,57]. The competitive NOS inhibitor l-NMMA has been employed in EAE, with variable efficacy, and treatment with the O_2_^−^ scavenger *N*-AC suppressed acute EAE through biasing immune response mechanisms [58–61]. In the present investigation the addition of UA, l-NMMA and *N*-AC to b.End3 cultures effectively reduced the spontaneous and glutamate-induced release of ONOO^−^ and therefore indicates a common target for the action of the compounds in EAE.

The identity of the mechanisms controlling ONOO^−^ production by b.End3 cells becomes clearer with the use of NMDA and AMPA/kainate receptor antagonists. We and others have confirmed the inhibitory influence of the NMDA receptor antagonists MK-801 and memantine on the neurological course and BBB disruption observed in EAE [12,13]. Furthermore, the usefulness of the AMPA/kainate receptor blocker NBQX has been shown in acute and chronic-relapsing models of the disease [14,15,20]. Interestingly, the generation of ONOO^−^, by the endothelial cell line, was exclusively inhibited by the NMDA receptor antagonists MK-801 and memantine. Involvement of the NMDA receptor in ONOO^−^ formation was further confirmed by the release of the molecule following incubation of cultures with the specific agonist NMDA. Finally, the presence of the receptor in association with b.End3 cells was demonstrated by the constitutive expression of NMDA R1 and NMDA R2A/B receptors, as has been previously noted for cerebral endothelial cells [62].

Results from the current study strongly indicating NMDA receptor presence on b.End3 cells, via the use of ionotropic and metabotropic receptor antagonists, are comparable to previous observations made using human and immortalized brain endothelial cells [27]. In particular, exposure of the human-derived cultures to glutamate or NMDA reduced endothelial barrier function that could be blocked using MK-801 and N-AC. Therefore, the data clearly indicate the presence of NMDA receptors in neuroendothelial preparations and involvement of the receptor in endothelial function. Also, both studies add credence to the theory that the monosodium derivative of glutamate may be toxic at neurovascular sites and lead to a breakdown in normal BBB function [63,64].

Glutamate-induced NMDA receptor activation leads to removal of the magnesium channel block, calcium influx and the up-regulation of various enzymes including NOS [9]. NOS exists as several isoforms that are susceptible to inhibition by specific and non-specific agents [65]. The preferential reduction of ONOO^−^ production by the selective eNOS inhibitor l-NIO strongly suggests involvement of the isoform in glutamate receptor-mediated events. Moreover, exposure of cells to the specific iNOS inhibitor 1400W [66,67] was without effect on ONOO^−^ production indicating no role for the isoform in generation of the free radical. Therefore, glutamate may act at the NMDA receptor site on brain endothelial cells to enhance calcium influx thereby up-regulating eNOS, to cause the formation of NO and the subsequent generation of ONOO^−^.

In conclusion, the study has shown the brain-derived cell line b.End3 produces NO and ONOO^−^ in response to non-cytotoxic concentrations of the amino acid glutamate. The work also provides clear evidence that glutamate promotes inflammatory mediator release by acting as an agonist for b.End3-associated NMDA receptors. The results offer more understanding of the important relationship between glutamate and the NMDA receptor positioned at neurovascular sites that may be of particular relevance in the pathogenesis of demyelinating disease and other CNS-related conditions.

## References

[1] Groom A.J., Smith T., Turski L. (2003). Multiple sclerosis and glutamate. Ann NY Acad Sci.

[2] Killestein J., Kalkers N.F., Polman C.H. (2005). Glutamate inhibition in MS: the neuroprotective properties of riluzole. J Neurol Sci.

[3] Sastre-Garriga J., Ingle G.T., Chard D.T., Ramio-Torrenta L., McLean M.A., Miller D.H. (2005). Metabolite changes in normal-appearing gray and white matter are linked with disability in early primary progressive multiple sclerosis. Arch Neurol.

[4] Stover J.F., Pleines U.E., Morganti-Kossmann M.C., Kossmann T., Lowitzsch K., Kempski O.S. (1997). Neurotransmitters in cerebrospinal fluid reflect pathological activity. Eur J Pathol Invest.

[5] Sarchielli P., Greco L., Floridi A., Floridi A., Gallai V. (2003). Excitatory amino acids and multiple sclerosis: evidence from cerebrospinal fluid. Arch Neurol.

[6] Srinivasan R., Sailasuta N., Hurd R., Nelson S., Pelletier D. (2005). Evidence of elevated glutamate in multiple sclerosis using magnetic resonance spectroscopy at 3 T. Brain.

[7] Vallejo-Illarramendi A., Domercq M., Perez-Cerda F., Ravid R., Matute C. (2006). Increased expression and function of glutamate transporters in multiple sclerosis. Neurobiol Dis.

[8] Werner P., Pitt D., Raine C.S. (2001). Multiple sclerosis: altered glutamate homeostasis in lesions correlates with oligodendrocyte and axonal damage. Ann Neurol.

[9] Bolton C., Paul C. (2006). Glutamate receptors in neuroinflammatory demyelinating disease. Med Inflamm.

[10] Hardin-Pouzet H., Krakowski M., Bourbonniere L., Didier-Bazes M., Tran E., Owens T. (1997). Glutamate metabolism is down-regulated in astrocytes during experimental allergic encephalomyelitis. Glia.

[11] Ohgoh M., Hanada T., Smith T., Hashimoto T., Ueno M., Yamanishi Y. (2002). Altered expression of glutamate transporters in experimental autoimmune encephalomyelitis. J Neuroimmunol.

[12] Bolton C., Paul C. (1997). MK-801 limits neurovascular dysfunction during experimental allergic encephalomyelitis. J Pharmacol Exp Ther.

[13] Paul C., Bolton C. (2002). Modulation of blood–brain barrier dysfunction and neurological deficits during acute experimental allergic encephalomyelitis by the *N*-methyl-d-aspartate receptor antagonist memantine. J Pharmacol Exp Ther.

[14] Smith T., Groom A., Zhu B., Turski L. (2000). Autoimmune encephalomyelitis ameliorated by AMPA antagonists. Nat Med.

[15] Pitt D., Werner P., Raine C.S. (2000). Glutamate excitotoxicity in a model of multiple sclerosis. Nat Med.

[16] Gilgun-Sherki Y., Panet H., Melamed E., Offen D. (2003). Riluzole suppresses experimental autoimmune encephalomyelitis: implications for the treatment of multiple sclerosis. Brain Res.

[17] Meldrum B.S. (2000). Glutamate as a neurotransmitter in the brain: review of physiology and pathology. J Nutr.

[18] Geurts J.J., Wolswijk G., Bo L., van der Valk P., Polman C.H., Troost D. (2003). Altered expression patterns of group I and II metabotropic glutamate receptors in multiple sclerosis. Brain.

[19] Matute C., Domercq M., Sanchez-Gomez M.V. (2006). Glutamate-mediated glial injury: mechanisms and clinical importance. Glia.

[20] Kanwar J.R., Kanwar R.K., Krissansen G.W. (2004). Simultaneous neuroprotection and blockade of inflammation reverses autoimmune encephalomyelitis. Brain.

[21] Choi D. (1998). Antagonizing excitotoxicity: a therapeutic strategy for stroke?. Mt Sinai J Med.

[22] Hawkins B.T., Davis T.P. (2005). The blood–brain barrier/neurovascular unit in health and disease. Pharmacol Rev.

[23] Minagar A., Alexander J.S. (2003). Blood–brain barrier disruption in multiple sclerosis. Mult Scler.

[24] Ransohoff R.M., Kivisakk P., Kidd G. (2003). Three or more routes for leukocyte migration into the central nervous system. Nat Rev Immunol.

[25] Mayhan W.G., Didion S.P. (1996). Glutamate-induced disruption of the blood–brain barrier in rats: role of nitric oxide. Stroke.

[26] Collard C.D., Park K.A., Montalto M.C., Alapati S., Buras J.A., Stahl G.L. (2002). Neutrophil-derived glutamate regulates vascular endothelial barrier function. J Biol Chem.

[27] Sharp C.D., Hines I., Houghton J., Warren A., Jackson T.H., Jawahar A. (2003). Glutamate causes a loss in human cerebral endothelial barrier integrity through the activation of the *N*-methyl-d-aspartate receptor (NMDA). Am J Physiol Heart Circ Physiol.

[28] Hooper D.C., Scott G.S., Zborek A., Mikheeva T., Kean R.B., Koprowski H. (2000). Uric acid, a peroxynitrite scavenger, inhibits CNS inflammation, blood–CNS barrier permeability changes, and tissue damage in a mouse model of multiple sclerosis. FASEB J.

[29] Kean R.B., Spitsin S.V., Mikheeva T., Scott G.S., Hooper D.C. (2000). The peroxynitrite scavenger uric acid prevents inflammatory cell invasion into the CNS in experimental allergic encephalomyelitis through maintenance of blood–CNS barrier integrity. J Immunol.

[30] Kastenbauer S., Koedel U., Pfister H.W. (1999). Role of peroxynitrite as a mediator of pathophysiological alterations in experimental pneumococcal meningitis. J Infect Dis.

[31] Gunasekar P.G., Kanthasamy A.G., Borowitz J.L., Isom G.E. (1995). NMDA receptor activation produces concurrent generation of nitric oxide and reactive oxygen species: implication for cell death. J Neurochem.

[32] Montesano R., Pepper M.S., Mohle-Steinlein U., Risau W., Wagner E.F., Orci L. (1990). Increased proteolytic activity is responsible for the aberrant morphogenetic behavior of endothelial cells expressing the middle T oncogene. Cell.

[33] Green L.C., Wagner D.A., Glogowski J., Skipper P.L., Wishnok J.S., Tannenbaum S.R. (1982). Analysis of nitrate and [^15^N] nitrate in biological fluids. Anal Biochem.

[34] Bogaert L., Scheller D., Moonen J., Sarre S., Smolders I., Ebinger G. (2000). Neurochemical changes and laser Doppler flowmetry in the endothelin-1 rat model for focal cerebral ischemia. Brain Res.

[35] Fujiwara M., Egashira N. (2004). New perspectives in the studies on endocannabinoid and cannabis: abnormal behaviors associate with CB1 cannabinoid receptor and development of therapeutic application. J Pharmacol Sci.

[36] Parfenova H., Basuroy S., Bhattacharya S., Tcheranova D., Qu Y., Regan R.F. (2006). Glutamate induces oxidative stress and apoptosis in cerebral vascular endothelial cells: contributions of HO-1 and HO-2 to cytoprotection. Am J Physiol Cell Physiol.

[37] Kew J.N., Kemp J.A. (2005). Ionotropic and metabotropic glutamate receptor structure and pharmacology. Psychopharmacology.

[38] Chazot P.L., Stevenson F.A. (1997). Biochemical evidence for the existence of a pool of unassembled C2 exon-containing NR1 subunits of the mammalian forebrain. J Neurochem.

[39] Moon I.S., Apperson M.L., Kennedy M.B. (1994). The major tyrosine-phosphorylated protein in the postsynaptic density fraction is *N*-methyl-d-aspartate receptor subunit 2B. Proc Natl Acad Sci USA.

[40] Rees D.D., Palmer R.M., Schulz R., Hodson H.F., Moncada S. (1990). Characterization of three inhibitors of endothelial nitric oxide synthase in vitro and in vivo. Br J Pharmacol.

[41] Hallinan E.A., Tsymbalov S., Dorn C.R., Pitzele B.S., Hansen D.W., Moore W.M. (2002). Synthesis and biological characterization of L-*N*(6)-(1-iminoethyl)lysine 5-tetrazole-amide, a prodrug of a selective iNOS inhibitor. J Med Chem.

[42] Garvey E.P., Oplinger J.A., Furfine E.S., Kiff R.J., Laszlo F., Whittle B.J. (1997). 1400W is a slow, tight binding, and highly selective inhibitor of inducible nitric-oxide synthase in vitro and in vivo. J Biol Chem.

[43] Castelijns J.A., Barkhof F. (1999). Magnetic resonance (MR) imaging as a marker for multiple sclerosis. Biomed Pharmacother.

[44] Petty M.A., Lo E.H. (2002). Junctional complexes of the blood–brain barrier: permeability changes in neuroinflammation. Prog Neurobiol.

[45] Lo E.H., Wang X., Cuzner M.L. (2002). Extracellular proteolysis in brain injury and inflammation: role for plasminogen activators and matrix metalloproteinases. J Neurosci Res.

[46] Encinas J.M., Manganas L., Enikolopov G. (2005). Nitric oxide and multiple sclerosis. Curr Neurol Neurosci Rep.

[47] Scott G.S., Kean R.B., Fabis M.J., Mikheeva T., Brimer C.M., Phares T.W. (2004). ICAM-1 upregulation in the spinal cords of PLSJL mice with experimental allergic encephalomyelitis is dependent upon TNF-alpha production triggered by the loss of blood–brain barrier integrity. J Neuroimmunol.

[48] Williams R.L., Risau W., Zerwes H.G., Drexler H., Aguzzi A., Wagner E.F. (1989). Endothelioma cells expressing the polyoma middle T oncogene induce hemangiomas by host cell recruitment. Cell.

[49] Wagner E.F., Risau W. (1994). Oncogenes in the study of endothelial cell growth and differentiation. Semin Cancer Biol.

[50] Williams R.L., Courtneidge S.A., Wagner E.F. (1988). Embryonic lethalities and endothelial tumors in chimeric mice expressing polyoma virus middle T oncogene. Cell.

[51] Bocchietto E., Guglielmetti A., Silvagno F., Taraboletti G., Pescarmona G.P., Mantovani A. (1993). Proliferative and migratory responses of murine microvascular endothelial cells to granulocyte-colony-stimulating factor. J Cell Physiol.

[52] Garlanda C., Parravicini C., Sironi M., De Rossi M., Wainstok de Calmanovici R. (1994). Progressive growth in immunodeficient mice and host cell recruitment by mouse endothelial cells transformed by polyoma middle-sized T antigen: implications for the pathogenesis of opportunistic vascular tumors. Proc Natl Acad Sci USA.

[53] Bussolino F., De Rossi M., Sica A., Colotta F., Wang J.M., Bocchietto E. (1991). Murine endothelioma cell lines transformed by polyoma middle T oncogene as target for and producers of cytokines. J Immunol.

[54] Yen T.C., Harrison C.A., Devery J.M., Leong S., Iismaa S.E., Yoshimura T. (1997). Induction of S100 chemotactic protein, CP-10, in murine microvascular endothelial cells by proinflammatory stimuli. Blood.

[55] Ghigo D., Arese M., Todde R., Vecchi A., Silvagno F., Costamagna C. (1995). Middle T antigen-transformed endothelial cells exhibit an increased activity of nitric oxide synthase. J Exp Med.

[56] Song L., Pachter J.S. (2003). Culture of murine brain microvascular endothelial cells that maintain expression and cytoskeletal association of tight function-associated proteins. In Vitro Cell Devlop Biol.

[57] Scott G.S., Spitsin S.V., Kean R.B., Mikheeva T., Koprowski H., Hooper D.C. (2002). Therapeutic intervention in experimental allergic encephalomyelitis by administration of uric acid precursors. Proc Natl Acad Sci USA.

[58] Zielasek J., Jung S., Gold R., Liew F.Y., Toyka K.V., Hartung H.P. (1995). Administration of nitric oxide synthase inhibitors in experimental autoimmune neuritis and experimental autoimmune encephalomyelitis. J Neuroimmunol.

[59] Scott G.S., Williams K.I., Bolton C. (1996). A pharmacological study on the role of nitric oxide in the pathogenesis of experimental allergic encephalomyelitis. Inflamm Res.

[60] Lehmann D., Karussis D., Misrachi-Koll R., Shezen E., Ovadia H., Abramsky O. (1994). Oral administration of the oxidant-scavenger *N*-acetyl-l-cysteine inhibits acute experimental autoimmune encephalomyelitis. J Neuroimmunol.

[61] Stanislaus R., Gilg A.G., Singh A.K., Singh I. (2005). *N*-Acetyl-l-cysteine ameliorates the inflammatory disease process in experimental autoimmune encephalomyelitis in Lewis rats. J Autoimmune Dis.

[62] Krizbai I.A., Deli M.A., Pestenacz A., Siklos L., Szabo C.A., Andras I. (1998). Expression of glutamate receptors on cultured cerebral endothelial cells. J Neurosci Res.

[63] Olney J.W., Ho O.L. (1970). Brain damage in infant mice following oral intake of glutamate, aspartate or cysteine. Nature.

[64] Olney J.W. (1984). Excitotoxins in foods. Neurobehav Toxicol Teratol.

[65] Li H., Poulos T.L. (2005). Structure-function studies on nitric oxide synthases. J Inorg Biochem.

[66] Menchen L.A., Colon A.L., Moro M.A., Leza J.C., Lizasoain I., Menchen P. (2001). *N*-(3-(amino methyl) benzyl) acetamidine, an inducible nitric oxide synthase inhibitor, decreases colonic inflammation induced by trinitrobenzene sulphuric acid in rats. Life Sci.

[67] Cheng X., Cheng X.S., Kuo K.H., Pang C.C. (2004). Inhibition of iNOS augments cardiovascular action of noradrenaline in streptozotocin-induced diabetes. Cardiovasc Res.

